# Multielement analysis of plant extracts with potential use in the treatment of peptic ulcers by synchrotron radiation total reflection X-ray fluorescence

**DOI:** 10.7717/peerj.5375

**Published:** 2018-09-12

**Authors:** Leticia Diniz Vieira, Káthia Takeda da Silva, Rodrigo Sanchez Giarola, Guilherme Franco Inocente, Hélio Kushima, Clelia Akiko Hiruma Lima, Joel Mesa Hormaza

**Affiliations:** 1 Departamento de Física e Biofísica, Instituto de Biociências de Botucatu, Universidade Estadual Paulista, Botucatu, São Paulo, Brazil; 2 Departamento de Farmacologia, Instituto de Biociências de Botucatu, Universidade Estadual Paulista, Botucatu, São Paulo, Brazil; 3 Departamento de Fisiologia, Instituto de Biociências de Botucatu, Universidade Estadual Paulista, Botucatu, São Paulo, Brazil

**Keywords:** *Alchornea glandulosa*, *Davilla nitida*, *Davilla elliptica*, Synchrotron radiation, Multielement analysis, Plant extracts

## Abstract

Some plants popularly employed for the treatment of peptic ulcers have proved to be attractive sources of new drugs. Despite extensive research, the pharmacological and toxicological potentials of these plants are not fully understood. In this context, the aim of this work was to analyze the multielemental composition of the methanolic extracts of three of those plants, *Alchornea glandulosa* (AG), *Davilla elliptica* (DE) and *Davilla nitida* (DN), with the intention of contributing to the understanding of the mechanisms of action of these extracts. For this purpose, we used the analytical technique of total reflection X-ray fluorescence (TXRF) by synchrotron radiation at the Brazilian Synchrotron Light Source (LNLS/CNPEM). It was possible to determine the concentrations of the elements: P, S, Cl, K, Ca, Ti, Cr, Mn, Fe, Ni, Cu, Zn, Rb and Br in all of the samples. Selenium (Se) was detected only in the DN extract. An inverse relationship between the concentrations of elements with proven effectiveness and the gastroprotective activity of extracts considering induction protocols with ethanol and non-steroidal anti-inflammatory drugs (NSAIDs) was obtained. This data suggests that the function of the extract is not only associated with providing the elements for restoring the gastric mucosa but that it also promotes the displacement of these elements from other parts of the mucosa to the damaged area. Correlations between the concentrations of the elements were also obtained. In the DE extract, which is the most effective extract for both induction protocols, the obtained correlations were above 70% among almost all of the elements, and no anticorrelations were found. For the other two extracts, in the less effective extract (AG) anticorrelations above 70% were predominantly found. Meanwhile, in the DN extract, a few high anticorrelations were found, which may explain its intermediate stage of effectiveness.

## Introduction

Peptic ulcers are lesions caused by the instability of harmful and protective factors of the gastric and duodenal mucosa. These lesions can occur in response to chronic stress, smoking, alcohol, anti-inflammatory drugs such as non-steroidal drugs (NSAIDs) and the presence of *Helicobacter pylori* in the gastrointestinal tract (Go, 1997; Kushima, 2006).

To treat this type of disease, plant extracts have yielded promising results (Lewis & Hanson, 1991; Borrelli & Izzo, 2000). An attractive source of new drugs, plant extracts are traditionally applied in popular medicine, playing an outstanding role in the protection of health as a preventive or supportive therapy in many populations. The therapeutic capacities of plants are mainly associated with the contents of biologically active organic compounds with a wide range of structures and curative powers, such as tannins, oils, alkaloids, flavonoids, and vitamins among others (Yamashita, 2006).

Two plants evaluated in this study are *Alchornea glandulosa* (AG) and *Davilla elliptica* (DE), which are popularly used in the treatment of peptic ulcers. *Davilla nitida* (DN) was also evaluated. This plant is not a popular treatment but has gastroprotective activity (Kushima et al., 2009).

AG (Euphorbiaceae) is a tree of 10–20 m that can be found in southern and southwestern Brazil (Lorenzi, 1998; Calvo et al., 2007). According to surveys, animals pretreated with the extract of this plant (500 mg/kg, p.o.) showed a 57% reduction in ethanol-induced gastric ulcers and a 34% reduction in NSAID-induced ulcers when compared to the control group. In this research, the antiulcerogenic effects of AG were associated with its compounds of quercetin and myricetin derivatives, the biflavonoid amentoflavone, gallic acid, methyl gallate, and the alkaloid pterogynidine (Calvo et al., 2007).

DE (Dilleniaceae) is a shrub up to 3-m tall found in places such as the Brazilian Cerrado (Silva et al., 2001; Kushima et al., 2009). According to ethnopharmacological research, leaves of DE are employed in folk medicine to treat diseases such as inflammation and ulcers. Pretreatment with this extract (500 mg/kg, p.o.) showed a 95% reduction in gastric ulcers induced by ethanol and a 77% reduction in NSAID-induced ulcers when compared to the control group (Kushima et al., 2009).

DN, also part of the Dilleniaceae family, is a climbing shrub that can reach 4 m found in the Brazilian Cerrado (Kushima et al., 2009). Although not used in folk medicine, studies with DN have shown that it has an equivalent chemical composition to DE, in which compounds involved in gastroprotective effects have been identified (Zayachkivska et al., 2005; Rodrigues et al., 2008; Kushima et al., 2009). Research with its extract (500 mg/kg, p.o.) also showed an 88% reduction in ethanol-induced gastric ulcers when compared to the control group. For NSAID-induced ulcers, a reduction of 67% was demonstrated (Kushima et al., 2009).

Even with these surveys, the understanding of the pharmacological and toxicological mechanisms of herbal compounds produced from these plants is not yet complete. Many of these studies were performed with organic constituents and few studies have shown the roles of inorganic elements in the medicinal use of these plants. Therefore, the purpose of this work was to study the multielemental analysis of the extracts of AG, DE and DN, evaluating the possible influence of their constituent chemical elements on the pharmacological effects of their extracts. We opted to focus on the quantitative determination of elements relevant for the understanding of the effectiveness and pharmacological action of the medicinal plants.

Due to the importance of the elements present in medicinal herbs, several studies have been carried out to determine their concentrations by using the following techniques: atomic absorption spectrometry (AAS) (Malik et al., 2008; Kolasani, Xu & Millikan, 2011; Ayivor et al., 2012; Malik et al., 2013), inductively coupled plasma-mass spectrometry (ICP-MS) (Tokalioğlu, 2012; Zhang et al., 2013), inductively coupled plasma-atomic emission spectrometry (ICP-AES) (Leśniewicz, Jaworska & Zyrnicki, 2006; Başgel & Erdemoğlu, 2006), inductively coupled plasma-optical emission spectrometry (ICP-OES) (Malik et al., 2008; Pytlakowska et al., 2012; Malik et al., 2013), neutron activation analysis (NAA) (Ayivor et al., 2012; Fatima, Waheed & Zaidi, 2013; Waheed & Fatima, 2013), particle induced X-ray emission (PIXE) (Naga Raju et al., 2006; Rihawy et al., 2010; Venkateswara Rao et al., 2013) and X-ray fluorescence (XRF) (Marguí, Hidalgo & Queralt, 2005; Khuder et al., 2009; Hondrogiannis et al., 2012; De La Calle et al., 2013).

Total reflection X-ray fluorescence (TXRF) spectrometry is a version of the XRF and a recognized tool for elemental trace and microanalysis (Klockenkämper, 1997). The major advantage of this analytical technique when compared to others is the need for a very small amount of sample, and that it can also be used for the direct analysis of biological samples (Szoboszlai et al., 2009). Moreover, TXRF is a simultaneous technique with very low detection limits and easy patterning with an internal standard element (Klockenkämper, 1997; Szoboszlai et al., 2009). To increase the performance of the TXRF, synchrotron radiation (SR) is used as a source of X-rays. This provides X-rays with a high intensity and broad energy range for the technique (Kang et al., 2002; Meirer et al., 2010).

In this work, the SR-TXRF method was used to determine the concentrations of various major and trace elements in the extracts of AG, DE and DN. The 15 analyzed elements were divided into macro-essential (K, Ca, S and P), micro-essential (Mn, Fe, Cu, Zn, Se, Ni, Cr, Cl and Br) and non-essential or toxic (Rb and Ti). The possible relationship between the concentrations of the elements and the effectiveness of each extract will also be discussed.

## Materials & Methods

### Sample preparation

The plant extracts were prepared by the Laboratory of Biological Assays with Natural Products, UNESP-Botucatu, SP. These medicinal plants are an important natural resource with a wide distribution and occurrence in Brazil, and no endangered or protected species were involved according to the Brazilian Institute of Environment and Renewable Natural Resources (IBAMA). Thus, no specific permissions were required for this work.

In the preparations, leaves of AG, DE and DN were extracted with methanol. Dry DE and DN leaves were extracted during one week and dry AG leaves were extracted during 48 h, at room temperature. After the evaporation of the solvents, methanolic extracts of AG, DE and DN were obtained and maintained at 5 °C until the analysis (Calvo et al., 2007; Kushima et al., 2009).

For the quantitative determination of elemental SR-TXRF, gallium (Ga) was used as internal standard (5.125 mg L^−1^) because it was not present in the original sample and also because it allowed the correction of factors of the system. It was prepared from a 1.025 g L^−1^ Ga atomic absorption solution (Aldrich, Inc., St. Louis, MO, USA) using an appropriate dilution. An aliquot of 25 µL of Ga was added to 25 µL of each extract sample in a test tube. Then, 5 µL aliquots of this solution, in triplicate, were placed on the Si (1 1 1) substrates, followed by drying in an oven at 60 °C.

### SR-TXRF measurements

The measurements were performed at the X-ray fluorescence beam line of the Brazilian Synchrotron Light Laboratory (LNLS) (Pérez et al., 1999), Campinas (SP), Brazil. The excitation of the samples deposited on Si (1 1 1) layers was performed using synchrotron radiation of a beam operating at 16.5 keV. The fluorescence radiation was recorded by a Si(Li) detector with energy resolution of 165 eV at 5.9 keV, and the detector was placed at a 90°  angle to the incident beam (Pérez et al., 1999). The irradiation time was 100 s, and the X-ray spectra obtained were evaluated by the software AXIL (Bernasconi & Tajani, 1996) in order to obtain the X-ray intensities for each element and the associated uncertainty. The intensities were corrected to account for the dead time and the incident beam. All measurements were made in triplicate.

The calibration system and quantitative analysis depend on the determination of the sensitivity for each element. For this purpose, standard solutions (Multielement Atomic Spectroscopy Solution) from Fluka (Buchs, Switzerland) containing the elements K, Ca, Cr, Mn, Fe, Ni, Cu, Zn, Ga, and Sr (K-shell lines) were used at five different known concentrations with the internal standard (Ga, 9.32 ppm) also being added. These standards were detected for 100 s.

The common procedure for concentration determination by TXRF was adopted. From the calibration standards, the detection sensitivity *s*_*i*_ (cps mL µg^−1^) for each element was calculated by Eq. (1):

(1) \begin{eqnarray*}{s}_{i}= \frac{{I}_{i}}{{C}_{i}} \end{eqnarray*}

where *I*_*i*_ (cps) is the fluorescence intensity of element *i* and *C*_*i*_ (µg mL^−1^) is its concentration.

The same equation can be used to calculate the relationship between the X-ray intensity of the element of interest and the X-ray intensity emitted by the internal standard (Ga). This results in Eq. (2) to calculate the sensitivity of the system and to quantitatively analyze samples of unknown compositions:

(2) \begin{eqnarray*}\begin{array}{@{}l@{}} \displaystyle \frac{{s}_{i}}{{s}_{Ga}} = \frac{{I}_{i}}{{I}_{Ga}} \frac{{C}_{Ga}}{{C}_{i}} \vskip{6pt}\\ \displaystyle {R}_{i}= \frac{{I}_{i}}{{I}_{Ga}} {C}_{Ga}\vskip{6pt}\\ \displaystyle {S}_{i}= \frac{{s}_{i}}{{s}_{Ga}} \vskip{6pt}\\ \displaystyle {C}_{i}= \frac{{R}_{i}}{{S}_{i}} \end{array}\end{eqnarray*}

where *I*_*Ga*_ is the intensity of the internal standard (Ga) in the sample (cps); *C*_*Ga*_ is the concentration of the internal standard (Ga) in the sample (µg mL^−1^); and *s*_*Ga*_ is the sensitivity of the internal standard.

Detection limits for each element were determined using certified samples of Trace elements in natural water (NIST 1640) and Eq. (3):

(3) \begin{eqnarray*}LD=3\sqrt{ \frac{{I}_{BG}}{t} } \frac{{C}_{Ga}}{{I}_{Ga}{S}_{i}} \end{eqnarray*}

where *I*_*Bg*_ is the area below the fluorescent peak for the element *I* (Curie, 1968; Ladisich et al., 1993)*.* The values were calculated for the standard solution and were extrapolated to 1,000 s of measurement time.

### Statistical analysis

The extracts samples (*n* = 3) were analyzed in triplicate and the results were expressed as a mean  ± standard deviation. Student’s *t* test was used for comparison between two samples, and analysis of variance (ANOVA) was used for comparison among more than two samples. After ANOVA, the analysis was complemented by the multiple comparison Tukey–Kramer test using the significance level of 5%. Statistical calculations were performed using the InStat program, version 3.02 (GraphPad Software, San Diego California, USA).

The Pearson correlations between pairs of elemental concentrations were also calculated to identify possible synergies between them. For correlation analysis, it is common to associate values greater than 70% (in absolute values) with a high correlation (or anticorrelation in the negative case) between variables.

## Results

**Figure 1:**
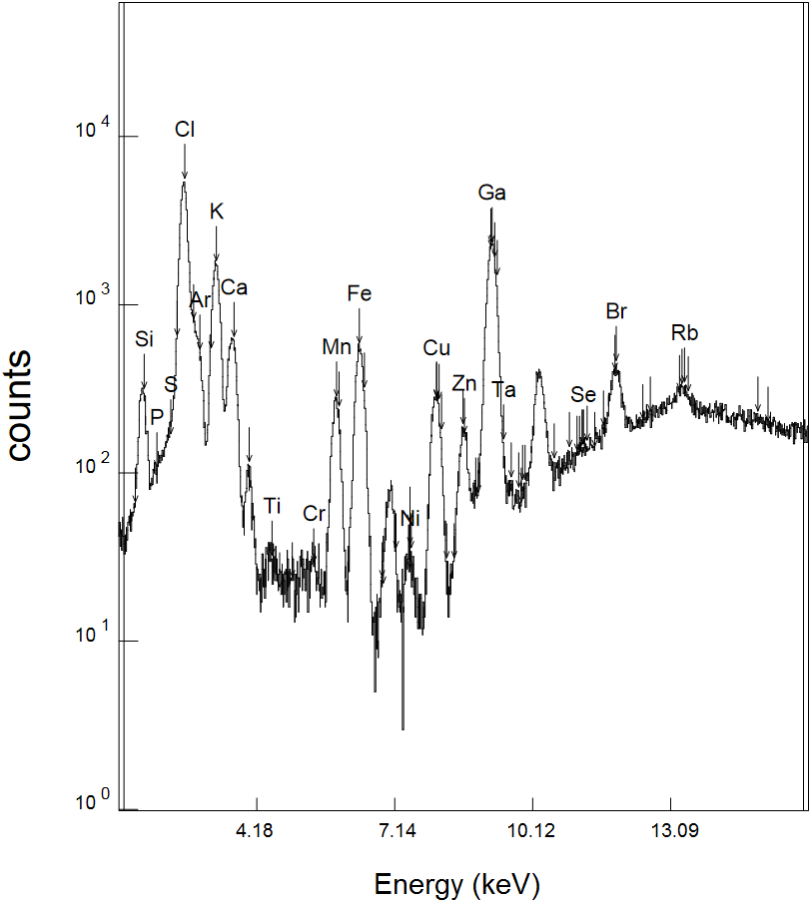
SR-TXRF spectrum (K*α* peaks) of a *Davilla elliptica* sample.

To evaluate the compositions of herbal extracts from plants used in the treatment of ulcers, elemental concentrations in the extracts were obtained by SR-TXRF. A typical spectrum as shown in Fig. 1 shows the presence of the elements P, S, Cl, K, Ca, Ti, Cr, Mn, Fe, Ni, Cu, Zn, Se, Br, and Rb.

**Figure 2:**
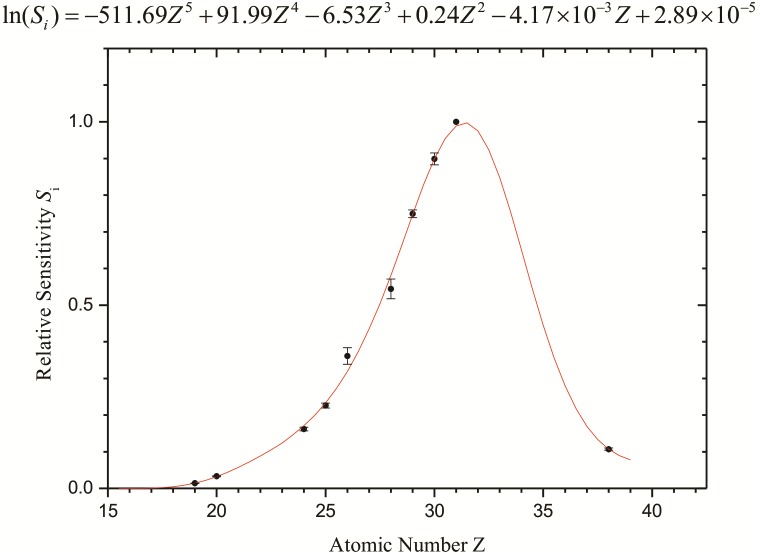
Relative sensitivities (internal standard: Ga) calculated for the elements. Error bars represent the standard deviation of samples.

For the quantitative analysis, the relative sensitivities of the elements were calculated using different dilutions of the standard solution containing the element Ga, which was also used as an internal standard in the sample. Thus, the relative sensitivity curve as a function of the atomic number for the K-shell line was determined (Fig. 2).

The error bars indicate the statistical and experimental detection limit uncertainties from the analysis of the elemental standard solutions in triplicate. The maximum of the sensitivity curve is for the element Ga (*Z* = 31), which has been used as an internal standard.

**Figure 3:**
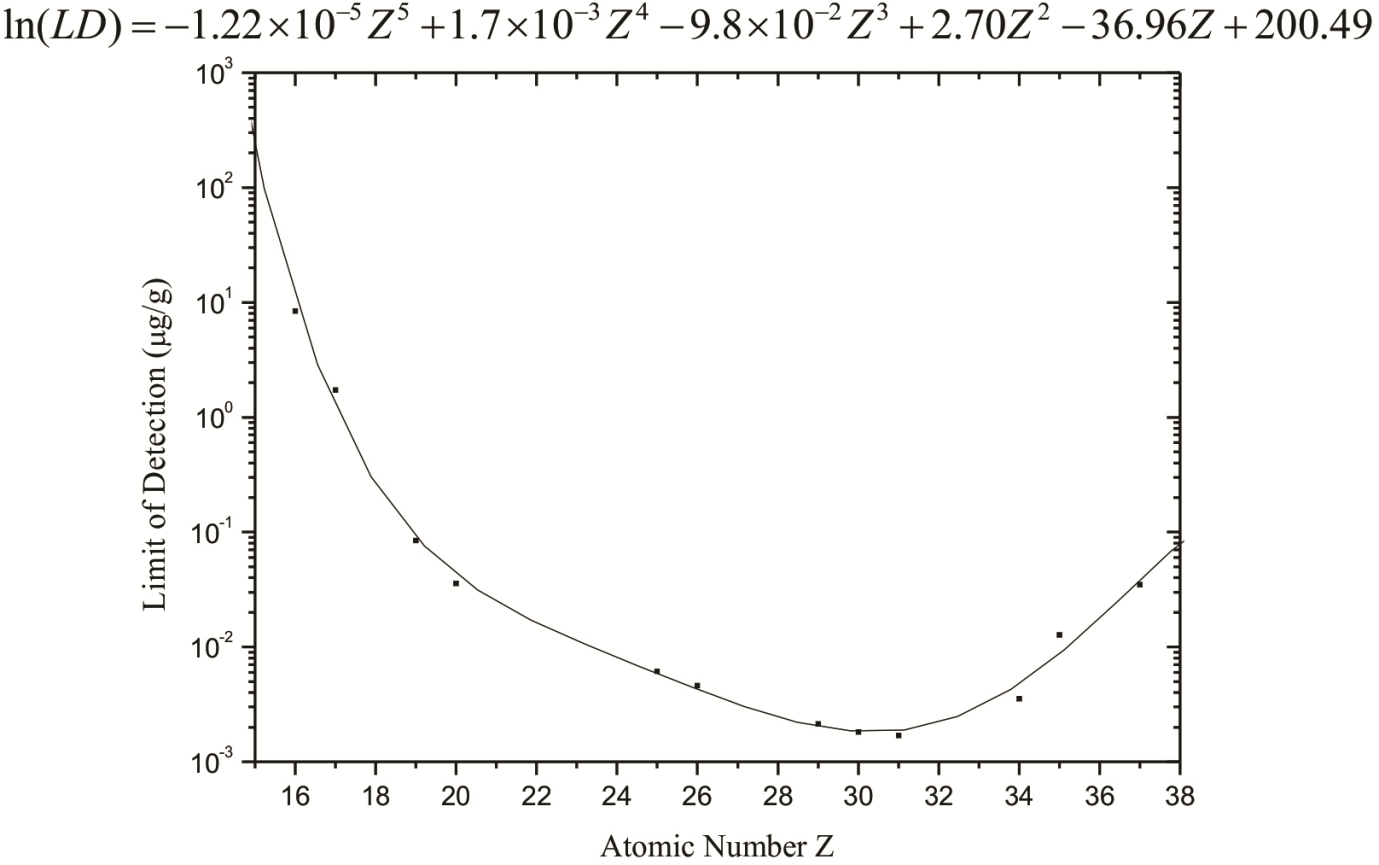
Limits of detection (µg g^−1^) calculated for the synchrotron radiation total reflection (SR-TXRF) system employed.

The limit of detection for each constituent element of the sample was also calculated from the spectra obtained by the standard samples. With these values, it was possible to determine the limit of detection as a function of the atomic number (Fig. 3).

Figure 3 shows that the detection limits obtained from Eq. (3) are at a level between 10^−3^ and 10^−1^ µg g^−1^ for elements heavier than K, but they increase significantly for lighter elements, reaching approximately 10 µg g^−1^ for S.

With the relative sensitivities obtained, the spectra of the samples were analyzed, and the concentrations of the elements were calculated based on the standard element (Table 1).

**Table 1:** Elemental concentration obtained (µg^−1^) for plants extracts analyzed by SR-TXRF (mean ± standard deviation).

Elements	Extracts		
	*Alchornea glandulosa*	*Davilla elliptica*	*Davilla nitida*
P (*Z* = 15)	136,529 ± 3,291^a^	32,270 ± 5,754^b^	45,494 ± 14,562^b^
S (*Z* = 16)	9,482 ± 27^a^	3,019 ± 375^b^	3,051 ± 796^b^
Cl (*Z* = 17)	23,730 ± 60^a^	9,180 ± 1,403^b^	9,500 ± 2,155^b^
K (*Z* = 19)	252.9 ± 0.2^a^	162 ± 24^a^	1,304 ± 324^b^
Ca (*Z* = 20)	182.7 ± 0.6^a^	21.2 ± 2.6^b^	74.4 ± 16.3^c^
Ti (*Z* = 22)	0.95 ± 0.04^a^	0.14 ± 0.03^b^	0.23 ± 0.02^b^
Cr (*Z* = 24)	0.23 ± 0.03^a^	0.20 ± 0.01^a^	0.25 ± 0.04^a^
Mn (*Z* = 25)	1.15 ± 0.02^a^	1.89 ± 0.17^a^	8.79 ± 2.21^b^
Fe (*Z* = 26)	10.54 ± 0.05^a^	2.28 ± 0.53^b^	3.24 ± 0.72^b^
Ni (*Z* = 28)	0.03 ± 0.01^a^	0.05 ± 0.01^b^	0.71 ± 0.16^b^
Cu (*Z* = 29)	1.38 ± 0.01^a^	0.83 ± 0.04^a^	1.16 ± 0.16^a^
Zn (*Z* = 30)	1.27 ± 0.01^a^	0.32 ± 0.03^a^	1.18 ± 0.28^a^
Se (*Z* = 34)	Nd	Nd	0.04 ± 0.01
Br (*Z* = 35)	0.59 ± 0.03^a^	1.12 ± 0.06^b^	1.13 ± 0.09^b^
Rb (*Z* = 37)	0.25 ± 0.05^a^	0.59 ± 0.15^a^	3.39 ± 0.59^b^

**Notes.**

a,b,c Different letters represent statistical differences (*p* < 0.05) between the concentrations of the same element in different samples. Nd: none determined element.

The elemental concentrations were in the range of 0.03 µg g^−1^ (Ni in AG extract) to 136,529 µg g^−1^ (P in AG extract). The concentrations of P, S, Cl, K and Ca were the highest, while those of Ni, Cr and Rb were the smallest. Se was detected only in the DN extract with a concentration of 0.04 µg g^−1^.

From the mean values of the element concentrations in the samples, it can be observed that P was the element with the highest concentration in all of the extracts followed by Cl, S, K and Ca, in descending order. By considering this set of elements (except K), higher concentrations of them were found in AG and smaller ones were found in DE. A statistically significant (*p* < 0.05) higher concentration of K (also Mn and Rb) was obtained in DN.

The AG samples showed higher values and statistical differences (*p* < 0.05) in the P, S, Cl, Ca, Ti and Fe concentrations when compared to DE and DN samples. Statistically significant lower concentrations of Ni and Br were also obtained.

No statistical differences were observed in the Cr, Cu and Zn contents.

Se, which is toxic at high concentrations (Terry et al., 2000), was only detected in the samples of DN but in low concentrations. Other toxic elements such as As, Cd, Pb and Hg were not detected.

In this way, besides obtaining the elemental concentrations of each extract, we also calculated the Pearson correlation coefficients between the concentrations of different elements for each type of sample.

For the DE extract (Table 2), the majority of the correlations are greater than 90% and positive. No anticorrelations greater than 70% were observed.

**Table 2:** Pearson correlation coefficient to *Davilla elliptica* extract. Higher correlations were highlighted in bold.

Elements	P	S	Cl	K	Ca	Ti	Cr	Mn	Fe	Ni	Cu	Zn	Br	Rb
P	1.000	—	–	–	–	–	–	–	–	–	–	–	–	–
S	**.974**	1.000	–	–	–	–	–	–	–	–	–	–	–	–
Cl	**.987**	**.998**	1.000	–	–	–	–	–	–	–	–	–	–	–
K	**.996**	**.990**	**.997**	1.000	–	–	–	–	–	–	–	–	–	–
Ca	**.974**	**.897**	**.925**	**.951**	1.000	–	–	–	–	–	–	–	–	–
Ti	**.999**	**.961**	**.978**	**.991**	**.984**	1.000	–	–	–	–	–	–	–	–
Cr	**.952**	**.858**	**.891**	**.923**	**.997**	**.967**	1.000	–	–	–	–	–	–	–
Mn	**.939**	**.837**	**.872**	**.907**	**.993**	**.956**	**.999**	1.000	–	–	–	–	–	–
Fe	**.932**	**.825**	**.862**	**.898**	**.990**	**.949**	**.998**	**1.000**	1.000	–	–	–	–	–
Ni	.518	.312	.376	.445	.699	.563	**.755**	**.781**	**.794**	1.000	–	–	–	–
Cu	−.452	−.642	−.588	−.526	−.237	−.405	−.158	−.118	−.097	.528	1.000	–	–	–
Zn	.353	.132	.199	.273	.557	.401	.622	.653	.669	**.983**	.675	1.000	–	–
Br	**.999**	**.978**	**.990**	**.998**	**.969**	**.998**	**.946**	**.933**	**.925**	.503	−.468	.336	1.000	–
Rb	.569	.368	.431	.498	**.742**	.612	**.793**	**.817**	**.829**	**.998**	.476	**.970**	.554	1.000

For the DN extract (Table 3), the majority of the correlations greater than 70% are positive. Anticorrelations greater than 70% were observed.

**Table 3:** Pearson correlation coefficient to *Davilla nitida* extract. Higher correlations (higher than 0.7) were highlighted in bold and higher anticorrelations (lower than −0.7) were highlighted in italic.

Elements	P	S	Cl	K	Ca	Ti	Cr	Mn	Fe	Ni	Cu	Zn	Se	Br	Rb
P	1.000	–	–	–	–	–	–	–	–	–	–	–	–	–	–
S	**.998**	1.000	–	–	–	–	–	–	–	–	–	–	–	–	–
Cl	**.985**	**.994**	1.000	–	–	–	–	–	–	–	–	–	–	–	–
K	**.995**	**.999**	**.997**	1.000	–	–	–	–	–	–	–	–	–	–	–
Ca	**.925**	**.948**	**.976**	**.959**	1.000	–	–	–	–	–	–	–	–	–	–
Ti	*−.974*	*−.958*	*−.922*	*−.947*	*−.816*	1.000	–	–	–	–	–	–	–	–	–
Cr	**.822**	**.857**	**.907**	**.875**	**.977**	−.673	1.000	–	–	–	–	–	–	–	–
Mn	**.939**	**.959**	**.984**	**.969**	**.999**	*−.838*	**.968**	1.000	–	–	–	–	–	–	–
Fe	.004	−.025	−.132	−.061	−.342	−.263	−.537	−.306	1.000	–	–	–	–	–	–
Ni	**.887**	**.915**	**.953**	**.929**	**.996**	*−.761*	**.992**	**.992**	−.426	1.000	–	–	–	–	–
Cu	*−.743*	*−.700*	−.617	−.671	−.433	**.874**	−.228	−.467	−.699	−.350	1.000	–	–	–	–
Zn	**.998**	**.992**	**.973**	**.987**	**.900**	*−.987*	**.784**	**.916**	.103	**.856**	*−.783*	1.000	–	–	–
Se	.241	.304	.403	.338	.591	−.017	**.751**	.560	*−.960*	.662	.471	.180	1.000	–	–
Br	**.941**	**.961**	**.985**	**.971**	**.999**	*−.841*	**.966**	**.999**	−.300	**.991**	−.473	**.918**	.555	1.000	–
Rb	**.995**	**.999**	**.997**	**.999**	**.958**	*−.948*	**.874**	**1.000**	−.058	**.928**	−.673	**.987**	.335	.335	1.000

For the AG extract (Table 4), the majority of the obtained correlations greater than 70% are negative but positive correlations were also observed.

**Table 4:** Pearson correlation coefficient to *Alchornea glandulosa* extract. Higher correlations (higher than 0.7) were highlighted in bold and higher anticorrelations (lower than −0.7) were highlighted in italic.

Elements	P	S	Cl	K	Ca	Ti	Cr	Mn	Fe	Ni	Cu	Zn	Br	Rb
P	1.000	–	–	–	–	–	–	–	–	–	–	–	–	–
S	**.763**	1.000	–	–	–	–	–	–	–	–	–	–	–	–
Cl	**.938**	.493	1.000	–	–	–	–	–	–	–	–	–	–	–
K	.543	**.957**	.218	1.000	–	–	–	–	–	–	–	–	–	–
Ca	**.894**	**.972**	.683	**.862**	1.000	–	–	–	–	–	–	–	–	–
Ti	.522	**.949**	.194	**1.000**	*−.849*	1.000	–	–	–	–	–	–	–	–
Cr	−.007	.640	−.353	**.836**	.442	**.849**	1.000	–	–	–	–	–	–	–
Mn	−.546	*−.958*	−.223	*−.998*	*−.864*	*−.999*	*−.834*	1.000	–	–	–	–	–	–
Fe	−.259	*−.821*	.092	*−.952*	−.665	*−.958*	*−.964*	**.950**	1.000	–	–	–	–	–
Ni	*−.898*	*−.970*	−.690	*−.857*	*−.972*	*−.844*	−.434	**.859**	.658	1.000	–	–	–	–
Cu	−.233	*−.806*	.118	*−.943*	−.654	*−.951*	*−.971*	**.942**	**.999**	.637	1.000	–	–	–
Zn	−.288	−.398	−.602	−.648	−.172	−.666	*−.960*	.644	**.850**	.163	**.864**	1.000	–	–
Br	−.002	−.636	.327	*−.851*	−.467	*−.863*	*−1.000*	**.848**	**.971**	.458	**.977**	**.951**	1.000	–
Rb	−.468	−.223	*−.745*	−.488	−.022	.509	**.887**	−.484	*−.732*	.031	*−.750*	*−.981*	*−.874*	1.000

## Discussion

In all analyzed extracts, we found essential nutrients such as Ca, Fe, K, P, Cr, Mn, Cu and Zn, which play a vital role in biochemical/enzymatic processes and participate as constituents of different antioxidant compounds (Fraga, 2005; Nielsen, 2009). Additionally, some of the elements have gastroprotective action (Watanabe et al., 1995; Marmiroli & Maestri, 2008). The observed variations in the elemental concentrations of the extracts can be attributed to differences in the botanical structures of the various plants, different compositions of the soil from which they were collected, the preferential absorbance of each plant species, the irrigation water and the climatic conditions (Queralt et al., 2005).

Among the detected elements, P, S, Cl, K, Ca, Fe, Mn, Cu and Zn are considered essential elements for plant growth because they are directly involved in nutrition, and their absence in plants hinders appropriate life cycles (Arnon & Stout, 1939; Prasad & Power, 1997). Although not considered essential elements, Ti, Cr, Ni and Rb show positive effects on plant growth. In the case of Br, even though it was detected in all of the samples, its relevance is not clear in this process (Kabata-Pendias & Pendias, 2001). For Se, despite much research regarding plants, its role is still controversial (Prasad & Power, 1997).

Independent of the form of consumption, the constituent elements of plants may be made available to the human body (Queralt et al., 2005). Thus, when there is deficiency of these elements in the diet, these plants can also be used for elemental supplementation (Daur, 2013).

After their incorporation, the elements can be classified as essential elements (P, S, Cl, K and Ca from the detected elements) and trace elements (Cr, Mn, Fe, Cu, Zn and Se from the detected elements). Although not all of the chemical elements possess biological activities that are fully described, most of them have functions directly related to the maintenance of health and the proper functioning of the body.

In literature there is no reported data for the multielemental analysis of AG, DE and DN. Therefore, the results obtained in the present study were compared with data from different plants traditionally employed in popular medicine.

Another species from the same genus of the AG, *Alchornea cordifolia* was analyzed by AAS and showed concentrations of Ca (288 mg kg^−1^), K (7.25 mg kg^−1^), Mn (58.35 mg kg^−1^), Fe (195.5 mg kg^−1^) and Cu (32.5 mg kg^−1^) (Alikwe & Owen, 2014). These concentrations are higher than any other obtained in this work, except for K.

Reported concentrations of Ca in leaves of the *Matricaria chamomilla* (189 mg kg^−1^) (Pytlakowska et al., 2012) and infusions of *Eucalyptus globulus* (24 mg kg^−1^) and *Matricaria chamomilla* (82 mg kg^−1^) (Queralt et al., 2005) are similar to those of the analyzed extracts in the present study. The element Ca has a role in the prevention and control of diseases (Desideri, Meli & Roselli, 2010) and was shown to interfere influence on cell proliferation and on the regeneration of damaged gastric mucosa (Koo, 1994).

The K levels in the extract of *Chelidonium majus* (262.5 mg kg^−1^) (Then et al., 2000), the infusion of *Salvia aucheri* (162.2 mg kg^−1^) (Özcan, 2005) and the leaves of *Hypericum calycinum* (1,295 µg g^−1^) (Pytlakowska et al., 2012) are similar to the K concentrations of AG, DE and DN, respectively. K is an element with an important role in the prevention and control of diseases (Desideri, Meli & Roselli, 2010).

The elements Mn and Fe are important for the metabolism of enzymes and also have immunomodulatory functions (Marmiroli & Maestri, 2008). The Fe concentrations in AG, DE and DN are similar to those reported for *Urtica dioica* (14.4 µg g^−1^) (Pytlakowska et al., 2012), *Camelia sinensis* (2.94 µg g^−1^) (Özcan et al., 2008) and *Hypericum calycinum* (4.61 µg g^−1^) (Pytlakowska et al., 2012). Our results for Mn in AG, DE and DN agreed with the ones reported by Özcan et al. (2008), laying between 0.40–125.74 µg g^−1^. The Mn concentrations were also reported a range of 1.2–188 mg kg^−1^ (Leal et al., 2013), 3.32–36.84 µg g^−1^ (Zegnin et al., 2008) and 3.65–397 µg g^−1^ (Salvador et al., 2003).

Reported concentrations of Cu in 20 turkey herbs were reported a range of 0.15–12.18 µg g^−1^ (Özcan et al., 2008). Our results for AG, DE and DN are within values usually reported in scientific literature. Cu is important for the functioning of many proteins and enzymes, and additionally, it has functions related to growth, blood cell production and the transport and metabolism of Fe. It also has the ability to interact with other elements, especially Zn, leading a competition for transporters (Marmiroli & Maestri, 2008).

The P levels in five medicinal plants commonly used in Spain analyzed by WDXRF (Queralt et al., 2005) and seven herbs consumed for medical purposes in Poland analyzed by ICP-OES (Pytlakowska et al., 2012) have been reported to range from 568 to 4,553 mg kg^−1^ and 1,652 to 5,731 µg g^−1^, respectively. In Brazil, the P levels in three different types of medicinal plants analyzed by TXRF (Salvador et al., 2003) were shown to vary from 1,540–13,100 µg g^−1^. Our results for AG, DE and DN are higher than the ones reported in other studies. The element P was detected in all of the extracts as expected. It participates in virtually all metabolic processes as part of structural molecules and as a component in enzymatic reactions (Nielsen, 2009), which are important for the growth and renewal of tissues (Desideri, Meli & Roselli, 2010).

The contents of S in DE and DN (3,019 µg g^−1^ and 3,051 µg g^−1^) are between values 160–8,200 µg g^−1^ reported by Salvador et al. (2003). Our result for AG (9,482 µg g^−1^) is higher than the one reported by the same study. S is an important element for humans due to its incorporation in amino acids, enzymes and other biomolecules. With the ability to synthesize compounds containing sulfur and the use of inorganic sulfur, plants become a relevant source of this element for humans (Komarnisky, Christopherson & Basu, 2003).

The results for Cl in the present study agree with the reported concentrations in five anti-diabetic medicinal plants analyzed by PIXE (7,200–48,400 µg g^−1^) (Naga Raju et al., 2006). Cl is one of the major ions in extracellular fluid, with activity related to acid–base balance and osmotic stability between cells. It is also responsible for muscle irritability, and it is present in the gastric juice in large quantities (Berdanier, 1994; Desideri, Meli & Roselli, 2010).

The concentrations of Ti in the present study are lower than those reported in literature. The Ti levels in *Helichrysi flos*, a herbal medicine in Europe (Lemberkovics et al., 2002), 8 anti-diabetic medicinal plants used in India (Naga Raju et al., 2006) and three medicinal plants used in Brazil (Salvador et al., 2003) laid between 1.3–2.8 mg kg^−1^, 18.7–68.9 µg g^−1^ and 5.44–177 µg g^−1^, respectively.

The Cr levels in AG, DE and DN are similar to the concentrations reported for tea of *Camellia sinensis* (0.22 µg g^−1^) (Mandiwana, Panichev & Panicheva, 2011), *Equisetum arvense* herb (0.20 mg kg^−1^) (Chizzola, 2012) and leaves of *Teucrium montanum* (0.263 µg g^−1^) (Cindric et al., 2013). Cr has a function associated with the action of insulin as well as carbohydrate, lipid and protein metabolism. Moreover, low concentrations of Cr may be related to cardiovascular disease (Marmiroli & Maestri, 2008). High concentrations of Cr in the air or in contact with the skin may induce chronic toxicity, but this toxicity as a result of oral ingestion is not very likely (WHO , 1996).

The contents of Ni in AG, DE and DN are similar to theose reported for *Urticadioica* infusion (0.04 µg g^−1^) (Başgel & Erdemoğlu, 2006) and *Sideritis* spp. herb (0.71 µg g^−1^) (Özcan et al., 2008; Chizzola, 2012).

In this study, Zn concentrations varied from 0.32 µg g^−1^ in DE to 1.27 µg g^−1^ in AG samples. The values reported in literature vary from 1.27–148 µg g^−1^ (Salvador et al., 2003), 1.8–10.6 mg kg^−1^ (Arsenijevic et al., 2011) and 14.9–39.4 µg g^−1^ (Cindric et al., 2013). Our results for DE and DN are lower than those reported in the previous studies. Zn is present in all of the tissues of the human body. It is also important as a component of various enzymes and for the metabolism of almost all macromolecules (Marmiroli & Maestri, 2008). Furthermore, Zn is an important factor in the healing of gastric lesions, especially in the initial phases (Watanabe et al., 1995).

Br levels in medicinal plants were reported a range of 0.72–142 mg kg^−1^ (Silva, Francisconi & Gonçalves, 2016), 2.51–12.3 µg g^−1^ (Khuder et al., 2009) and 5.4–26.8 µg g^−1^ (Naga Raju et al., 2006). Our results for AG are lower and the results for DE and DN are between reported values.

The contents of Rb in DN (3.39 µg g^−1^) are between values reported a range of 2.6–229 mg kg^−1^ (Silva, Francisconi & Gonçalves, 2016), 0.63–18.9 µg g^−1^ (Sussa et al., 2009) and 2.23–6.90 µg g^−1^ (Khuder et al., 2009). The concentrations in AG and DE samples are lower than the ones reported in the previous studies.

Se also presents effective gastroprotective effects and important metabolic functions (Kim et al., 2012). In literature, Se levels were reported a range of 0.602–0.964 µg g^−1^ (Kumar et al., 2005), 0.15–5.03 mg kg^−1^ (Özcan, 2004) and 1.65–23.53 mg kg^−1^ (Özcan et al., 2008). Leal et al. (2013) analyzed samples of 10 medicinal plants and only detected Se in *Paullinia cupana* (0.05 mg kg^−1^) and *Valeriana officinalis* (0.05 mg kg^−1^). In our results, Se was found only in DN samples and its concentration is lower than the ones reported previously.

As we can also see in Table 1, higher concentrations of the majority of elements with gastroprotective effects were found in the AG extract. From the extracts, AG presents a lower effectiveness (57% for ethanol-induced and 34% for NSAID-induced) compared with DE (95% for ethanol-induced and 77% for NSAID-induced) and DN (88% for ethanol-induced and 67% for NSAID-induced) (Calvo et al., 2007; Kushima et al., 2009). Thus, it is possible to establish that the role of the extracts is not only related to the direct deposition of these elements in the lesion but also to the promotion of their transport mechanisms from other regions of the gastric mucosa. Some of these mechanisms are associated with synergistic or antagonistic effects between two or more elements (Lokhande, Singare & Andhale, 2010; García-Barrera et al., 2012; Prasad, 2013).

From the results presented in Tables 2–4, we can infer that the effectiveness of the extracts may be linked in some way with the correlations obtained. Analyzing Table 2, we can only verify high correlations between elements in the extract of DE. This extract is the most effective for both induction protocols of gastric lesions (Calvo et al., 2007; Kushima et al., 2009).

High anticorrelations are predominant in the AG extract. In the case of Zn, an important element in the healing of gastric lesions, especially in the initial phases (Watanabe et al., 1995), and Ca, an element linked to cell proliferation and the regeneration of damaged gastric mucosa (Berdanier, 1994), the anticorrelations found could explain it having the lowest effectiveness. Meanwhile for the DN extract, a few high anticorrelations were found. Specifically, the result obtained for the Cu and Fe concentrations would justify its intermediate stage of effectiveness because of the synergetic function of Cu in the transport and metabolism of Fe, which is important for the metabolism of enzymes and also has immunomodulatory functions (Marmiroli & Maestri, 2008).

## Conclusions

Total reflection X-ray fluorescence with synchrotron radiation was successfully applied for the determination of the multielemental compositions of three phytotherapic extracts commonly used in folk medicine for the treatment of gastric diseases. An inverse relationship between the concentrations of the main elements with gastroprotective functions and the effectiveness of the extracts was obtained. Thus, it is possible to establish that the role of the extracts should not be related only to the direct deposition of these elements in the lesion but also with the promotion of their transport mechanisms from other regions of the gastric mucosa.

The predominant presence of the anticorrelations in the less effective extract (AG), specifically in the case of elements Zn and Ca that have proven gastroprotective function, could explain this extract having the lowest effectiveness. Meanwhile, in the DN extract, few high anticorrelations were obtained between concentrations, and those obtained were mainly for the elements Cu and Fe, which could explain this extract’s intermediate stage of effectiveness.

Although the direct relationship between the elemental concentrations in the extracts and their effectiveness is yet to be completed, the study of the chemical composition of plants is relevant to the understanding of their pharmacological and toxicological actions. In this work, the analysis demonstrated that the presence of essential elements may intensify the healing process in gastric diseases.

Consequently, further studies are being carried out by the authors to improve comprehension about the elemental contents of these extracts and their effectiveness by also considering the multielemental composition of gastric ulcers induced by NSAIDs and ethanol as well as their eventual modification in the presence of these phytotherapics.

##  Supplemental Information

10.7717/peerj.5375/supp-1 Table S1 Concentrations of the chemical elements in the analyzed extracts samples

## Additional Information and Declarations

### Data Availability

The following information was supplied regarding data availability: The raw data are provided in Table S1.
